# Safety and efficacy of short course combination regimens with AmBisome, miltefosine and paromomycin for the treatment of visceral leishmaniasis (VL) in Bangladesh

**DOI:** 10.1371/journal.pntd.0005635

**Published:** 2017-05-30

**Authors:** Ridwanur Rahman, Vishal Goyal, Rashidul Haque, Kazi Jamil, Abul Faiz, Rasheda Samad, Sally Ellis, Manica Balasegaram, Margriet den Boer, Suman Rijal, Nathalie Strub-Wourgaft, Fabiana Alves, Jorge Alvar, Bhawna Sharma

**Affiliations:** 1 Shaheed Suhrawardy Medical College (ShSMC), University of Dhaka, Dhaka, Bangladesh; 2 Drugs for Neglected Diseases *initiative* (DND*i*), Geneva, Switzerland; 3 International Centre for Diarrhoeal Disease Research (ICDDR,B), Dhaka, Bangladesh; 4 Kuwait institute for Scientific Research, Environment and Life Sciences Research centre, Food and Nutrition Program formerly ICDDR,B, Dhaka, Bangladesh; 5 Dev Care Foundation, Dhaka, Bangladesh and Retired Ministry of Health, Government official Dhaka, Bangladesh; 6 Chittagong Medical College, Chittagong, Bangladesh; Universidade Federal de Minas Gerais, BRAZIL

## Abstract

**Background:**

AmBisome therapy for VL has an excellent efficacy and safety profile and has been adopted as a first-line regimen in Bangladesh. Second-line treatment options are limited and should preferably be given in short course combinations in order to prevent the development of resistant strains. Combination regimens including AmBisome, paromomycin and miltefosine have proved to be safe and effective in the treatment of VL in India. In the present study, the safety and efficacy of these same combinations were assessed in field conditions in Bangladesh.

**Methods:**

The safety and efficacy of three combination regimens: a 5 mg/kg single dose of AmBisome + 7 subsequent days of miltefosine (2.5 mg/kg/day), a 5 mg/kg single dose of AmBisome + 10 subsequent days of paromomycin (15 mg/kg/day) and 10 days of paromomycin (15 mg/kg/day) + miltefosine (2.5 mg/kg/day), were compared with a standard regimen of AmBisome 15 mg/kg given in 5 mg/kg doses on days 1, 3 and 5. This was a phase III open label, individually randomized clinical trial. Patients from 5 to 60 years with uncomplicated primary VL were recruited from the Community Based Medical College Bangladesh (CBMC,B) and the Upazila Health Complexes of Trishal, Bhaluka and Fulbaria (all located in Mymensingh district), and randomly assigned to one of the treatments. The objective was to assess safety and definitive cure at 6 months after treatment.

**Results:**

601 patients recruited between July 2010 and September 2013 received either AmBisome monotherapy (n = 158), AmBisome + paromomycin (n = 159), AmBisome + miltefosine (n = 142) or paromomycin + miltefosine (n = 142). At 6 months post- treatment, final cure rates for the intention-to-treat population were 98.1% (95%CI 96.0–100) for AmBisome monotherapy, 99.4% (95%CI 98.2–100) for the AmBisome + paromomycin arm, 94.4% (95%CI 90.6–98.2) for the AmBisome + miltefosine arm, and 97.9% (95%CI 95.5–100) for paromomycin + miltefosine arm. There were 12 serious adverse events in the study in 11 patients that included 3 non-study drug related deaths. There were no relapses or PKDL up to 6 months follow-up. All treatments were well tolerated with no unexpected side effects. Adverse events were most frequent during treatment with miltefosine + paromomycin, three serious adverse events related to the treatment occurred in this arm, all of which resolved.

**Conclusion:**

None of the combinations were inferior to AmBisome in both the intention-to-treat and per-protocol populations. All the combinations demonstrated excellent overall efficacy, were well tolerated and safe, and could be deployed under field conditions in Bangladesh. The trial was conducted by the International Centre for Diarrhoeal Disease Research (ICDDR,B) and the Shaheed Suhrawardy Medical College (ShSMC), Dhaka, in collaboration with the trial sites and sponsored by the Drugs for Neglected Diseases *initiative* (DND*i*).

**Trial registration:**

ClinicalTrials.gov NCT01122771

## Author summary

Treatment is one of the key strategies for visceral leishmaniasis control and elimination. Historically a number of monotherapy drugs for VL treatment were used in Bangladesh, including pentavalent antimonials, amphotericin B deoxycholate (AmB), and miltefosine (MF). With the limited number of drugs available, it was necessary to preserve existing drugs and also to develop shorter and safer treatment regimens. At the time the study was initiated, miltefosine monotherapy was a recommended first-line treatment in Bangladesh. The present study aimed to provide safety and efficacy data for three short-course combination regimens including AmBisome, miltefosine and paromomycin when rolled out in field conditions in Bangladesh, and to compare these to AmBisome monotherapy. All combinations proved non-inferior to AmBisome monotherapy and were safe and well tolerated. This study was implemented in field conditions at Upazila level with treatment provided by government doctors, providing further evidence for scaling up new regimens in national program contexts within the public health sector.

## Introduction

### Background

World-wide, 200,000–400,000 new cases of visceral leishmaniasis (VL) occur annually [1]. The majority of these cases occur in South Asia; mainly in Bihar, India and neighbouring regions of Nepal, and in the highly endemic Mymensingh province of Bangladesh.

Before the introduction of single-dose AmBisome, miltefosine was included in the National Guidelines of India, Nepal and Bangladesh as a first line treatment for VL following a phase III trial in India that showed a final cure rate of 94% [2]. However, poor compliance due to its long treatment course (28 days) [3] and possible teratogenic effects have limited its successful roll-out. Moreover, a decrease in susceptibility to miltefosine was found in clinical isolates of relapse patients [4] and the efficacy of miltefosine declined to 90% within the last decade of use in South Asia [3]. A phase III clinical trial in India demonstrated that paromomycin at a dose of 15 mg/kg for 21 days provided a final cure rate of 94.6% [5], but this regimen was never implemented in Asia. As with miltefosine, there were indications that resistance to paromomycin might easily develop when used in monotherapy [6].

Due to the challenges in implementing clinical trials to assess treatment safety and efficacy, very few studies have been conducted in government sector at Upazila Health Complexes under real life conditions within national program settings. The present study aimed to assess the efficacy and safety of alternative combination treatments for primary visceral leishmaniasis and its implementation at different levels of the health system.

To reduce pressure on the drugs and prolong their therapeutic life-span, miltefosine and paromomycin should preferably be used in combination; short course combination regimens will lead to better compliance and are more readily implemented at health facility level. Three short-course combination regimens including AmBisome, miltefosine and paromomycin have been evaluated in a phase III clinical trial conducted in India (2008–2010). All showed an excellent safety profile and an efficacy of at least 97% in controlled conditions [7].

### Objective

The present study aimed to compare the safety and efficacy of the following combination regimens with AmBisome alone for the treatment of VL in Bangladesh:

- The combination of a single dose of AmBisome (day 1) with a 7 day course of miltefosine (day 2–8)
- The combination of a single dose of AmBisome (day 1) with a 10 day course of paromomycin (day 2–11)
- The combination of paromomycin with miltefosine (day 1–10)

## Methods

### Ethics statement

Ethical approval was obtained from the Ethical Review Committee of the International Centre for Diarrhoeal Disease Research, Bangladesh (ICDDR,B) and the National Research Ethics Committee (NREC) of the Bangladesh Medical Research Council (BMRC) prior to starting the study. The study was conducted in accordance with the ICH Harmonized Tripartite Guideline–Guideline for Good Clinical Practice (GCP), and ethical principles enshrined in the Declaration of Helsinki.

### Trial design and patients

This was a randomized, controlled, open-label, parallel group phase III clinical trial conducted in Mymensingh province, Bangladesh. Initially, 120 patients were recruited and treated in a hospital setting (the Community Based Medical College, CBMC) primarily for the purpose of confirming the safety of the combination treatments in Bangladesh. After review of safety data up to day 45 from the 120 patients treated at CBMC, and based on good safety profile of the treatments, the Data Safety Monitoring Board (DSMB) recommended to initiate recruitment at Upazila Health Complexes (UZHC). Additional 482 patients were recruited for the trial and treated in the Upazilas of Trishal, Gaffargoan and Bhaluka.

HIV negative primary VL patients between 5 and 60 years screened with positive rK39 rapid immunochromatographic tests (InBios, Seattle, USA) and parasitologically confirmed via bone marrow or spleen aspirates (only at CBMC) were enrolled into the study after giving informed consent. Because of the potential teratogenic effects of miltefosine, women of child-bearing age who were not using an assured method of contraception for the duration of treatment and three months afterwards were excluded, unless they agreed to receive an injection of medroxyprogesterone acetate (DepoProvera, Pfizer, NY, USA). One injection (which is effective for 3 months) was needed to ensure adequate coverage, taking into account miltefosine’s long half-life of approximately 7 days. Other exclusion criteria were: known hepatitis B, hepatitis C, or HIV infection, Hb concentrations less than 5 g/dl, platelet count of less than 40,000/mm^3^ (at CBMC only), a prothrombin time of more than 5 seconds longer than the control (at CBMC only), severe malnutrition [for adults (> 18 years) defined as BMI <14; for children (< 18 years) defined as BMI for age z score < -3 in children measuring > 121.5cm; and weight for height less than 60% in children measuring <121.5 cm], known alcohol or drug abuse, use of any investigational (unlicensed) drug within the last 3 months, and severe concurrent illnesses (TB, malaria) or chronic conditions (diabetes, hypertension). Pregnant and breast-feeding women, and patients with known hypersensitivity to the study drugs were also excluded.

### Treatment

Patients were randomized to four treatment arms. In the reference treatment group, patients were given the standard regimen of 15 mg/kg AmBisome, given in doses of 5 mg/kg on days 1, 3 and 5, infused over 2 hours in 5% dextrose solution. Patients allocated to the AmBisome + miltefosine arm received 5 mg/kg AmBisome on day 1, followed by 7 days of miltefosine (Impavido, Paladin, Canada). Miltefosine is provided as foil-wrapped blister packs of 10 and 50 mg capsules and was given to adults (>12 years) at a dose of 50 mg once daily if < 25 kg bodyweight, 50 mg twice daily if > 25 kg bodyweight; or 2.5 mg/kg/day divided into 2 doses for children younger than 12 years. Patients assigned to the AmBisome + paromomycin arm received 5 mg/kg AmBisome on day 1, followed by 10 days of paromomycin (Gland Pharma, India) at a dose of 11 mg/kg/day (base) equivalent to 15 mg/kg/day (sulphate salt), given by deep IM injection. Patients allocated to the miltefosine + paromomycin arm received the above described doses daily for 10 days. Patients treated with miltefosine on an outpatient basis in UZHC’s were given detailed instructions on the number of capsules to be taken each day, and asked to return to the clinic in case of vomiting. Miltefosine was re-dosed within one hour of vomiting. Patients were asked to return the empty blister packs for drug accountability.

### Outcomes

The main outcome was final cure, defined as initial cure at day 45 and absence of VL signs and symptoms during the follow-up period of 6 months.

The secondary outcome was initial cure, defined as clinical improvement at day 45. In the CBMC hospital setting, initial cure was confirmed by the absence of parasites in splenic/bone marrow aspirates at day 15. In cases of 1+ parasite at day 15, patients were retested at day 45. Patients who presented positive parasitology at day 45, or cases of relapse following combination treatment, were to receive rescue treatment with AmBisome 15 mg/kg. Those who failed to respond/relapsed following the reference treatment, or where AmBisome was contra-indicated, were to be rescued with sodium stibogluconate (SSG) 20mg/kg/day for 30 days, or miltefosine 2.5mg/kg/day for 28 days as recommended by the National Treatment Guidelines of Bangladesh and the investigator’s judgement. Patients with initial cure presenting with VL symptoms during follow-up were suspected of relapse, and were referred for parasitological confirmation at the CBMC hospital. Safety outcomes were adverse events and serious adverse events recorded during treatment and up to 6 months afterwards. Assessments were done on the basis of clinical adverse events systematically at all study sites. At the CBMC hospital, laboratory investigations included liver enzymes alanine aminotransferase (ALAT), aspartate aminotransferase (ASAT), bilirubin, prothrombin time, platelets, RBC count, WBC count, random blood glucose (RBS), urea, creatinine, serum sodium, potassium, magnesium. At the UZHC settings, laboratory parameters were limited to haemoglobin and random blood glucose (RBS). All patients were assessed for dermal manifestations of leishmaniasis (Para-KDL at baseline, and PKDL at day 15, 45 and at 6 months).

### Randomization

A computer generated randomization code was used for patient treatment allocation. Individual, opaque, sealed and sequentially numbered envelopes were provided to each study site (one envelope per patient), indicating the individual patient allocation to treatment. Eligible patients who fulfilled all the inclusion criteria, met none of the exclusion criteria and from whom informed consent had been obtained were randomized to treatment regimens using the sealed envelopes. Randomization was stratified by treatment centre and was done using an equal allocation ratio for married women, men, and children firstly; and further with an equal ratio in single women of childbearing potential. Single women of childbearing age were randomized to receive either AmBisome alone or AmBisome + paromomycin. The allocation ratio was adjusted to account for this, in order to achieve approximately equal numbers of patients in each arm. Thus the allocation ratio was 1:1:1:1 for four treatments in married women, men, and children; and 1:1 (for AmBisome alone or AmBisome + paromomycin) in single women of childbearing potential. This was an open-label study; miltefosine is an orally administered medication and AmBisome and paromomycin are administered IV and IM respectively.

### Sample size and study populations

The sample size was calculated assuming a treatment success of 97% in the reference arm (AmBisome) and a margin of non-inferiority of any tested treatment of 7%, leading to a minimally acceptable cure rate of 90% for each treatment. With a power of 90%, the sample size per group in the sample of married women, men and children would be 140. Based on the possible teratogenic effect of miltefosine and the assumption that 20% of the patients would be single women of child bearing potential, 26 extra patients were to be recruited among these women for both non-miltefosine groups. Assuming a drop-out rate of 10%, 154 patients were needed in both miltefosine groups and 183 in both non-miltefosine groups. The total sample size was calculated to be 674 patients.

The primary efficacy analysis was performed using the standard approach of non-inferiority for the comparison AmBisome vs AmBisome + miltefosine and for AmBisome vs. paromomycin + miltefosine on populations ITT1 and PP1. For AmBisome vs AmBisome + paromomycin comparisons, a logistic model was carried out using populations ITT and PP (Table 1).

**Table 1:** Definitive cure rate analysis: AmBisome monotherapy vs. comb. regimens.

Population	Treatment Group	Definitive Cure n (%)	Difference in proportion (%)	CI for difference in proportion (%)
**ITT population**	**AmB (N = 158)**	155 (98.1)	1.3	-1.73, 4.27
	**AmB + Paro (N = 159)**	158 (99.4)		

**PP population**	**AmB N = 156**	154 (98.7)	1.3	-0.88, 3.44
	**AmB + Paro N = 158**	158 (100.0)		

**ITT1 population**	**AmB N = 144**	141 (97.9)	-3.7	-9.20, 1.85
	**AmB + Milt N = 139**	131 (94.2)		

**PP1 population**	**AmB N = 142**	140 (98.6)	-3.2	-8.15, 1.80
	**AmB + Milt N = 131**	125 (95.4)		

**ITT1 population**	**AmB N = 144**	141 (97.9)	-0.1	-4.15, 4.03
	**Paro + Milt N = 140**	137 (97.9)		

**PP1 population**	**AmB N = 142**	140 (98.6)	-0.8	-4.60, 3.04
	**Paro + Milt N = 137**	134 (97.8)		

Denominator of the percentage is the number of patients in each treatment group.

The difference in the denominator of Ambisome arm between ITT (n = 158) and ITT1 (n = 144) is due to 14 single women of child-bearing age who were not included in the ITT1 for comparison with miltefosine arms.

The Intention to Treat 1 (ITT1) population included married women, men and children randomized to the trial and who received at least one dose of the study medication; but it did not include the single women of childbearing potential who were randomized either to AmBisome or Ambisome + paromomycin arms. The Intention to Treat population (ITT) included all patients randomised to the treatment groups who gave informed consent and who took at least one dose of study medication.

The Per Protocol 1 population (PP1) included patients (married women, men and children) enrolled in the Intent to Treat 1 population who were randomized to the trial, who had no major protocol deviations and who completed the 6 month follow-up visit or were classified as a treatment failure and received rescue medication. The Per Protocol population (PP) included all patients in the ITT population with no major protocol deviations and who completed the 6 month follow-up visit or were classified as a treatment failure and received rescue medication.

The ITT population was 601 and the PP population was 587. The reasons for exclusion in the PP population were 12 cases of withdrawal due to AE/SAE, which required rescue treatment, and 2 deaths.

## Results

### Patients

Of the 673 patients screened, 71 did not meet the inclusion criteria. A total of 24 screened patients did not have VL diagnosis confirmed by rK39 or microscopy. Among the patients with proven disease, the most common reasons for exclusion were chronic underlying disease (n = 14) and simultaneous participation in another study (n = 9). Five patients refused consent, four had abnormal laboratory parameters, 3 were pregnant or lactating women, and 12 were excluded for other reasons. The decision to stop recruitment was made after enrolling 602 patients (120 in the CBMC hospital setting, 482 in UZHC’s). Only 2% rather than the estimated 10% of patients were lost to follow-up, so that the sample size could be reduced by 72 patients.

**Fig 1:**
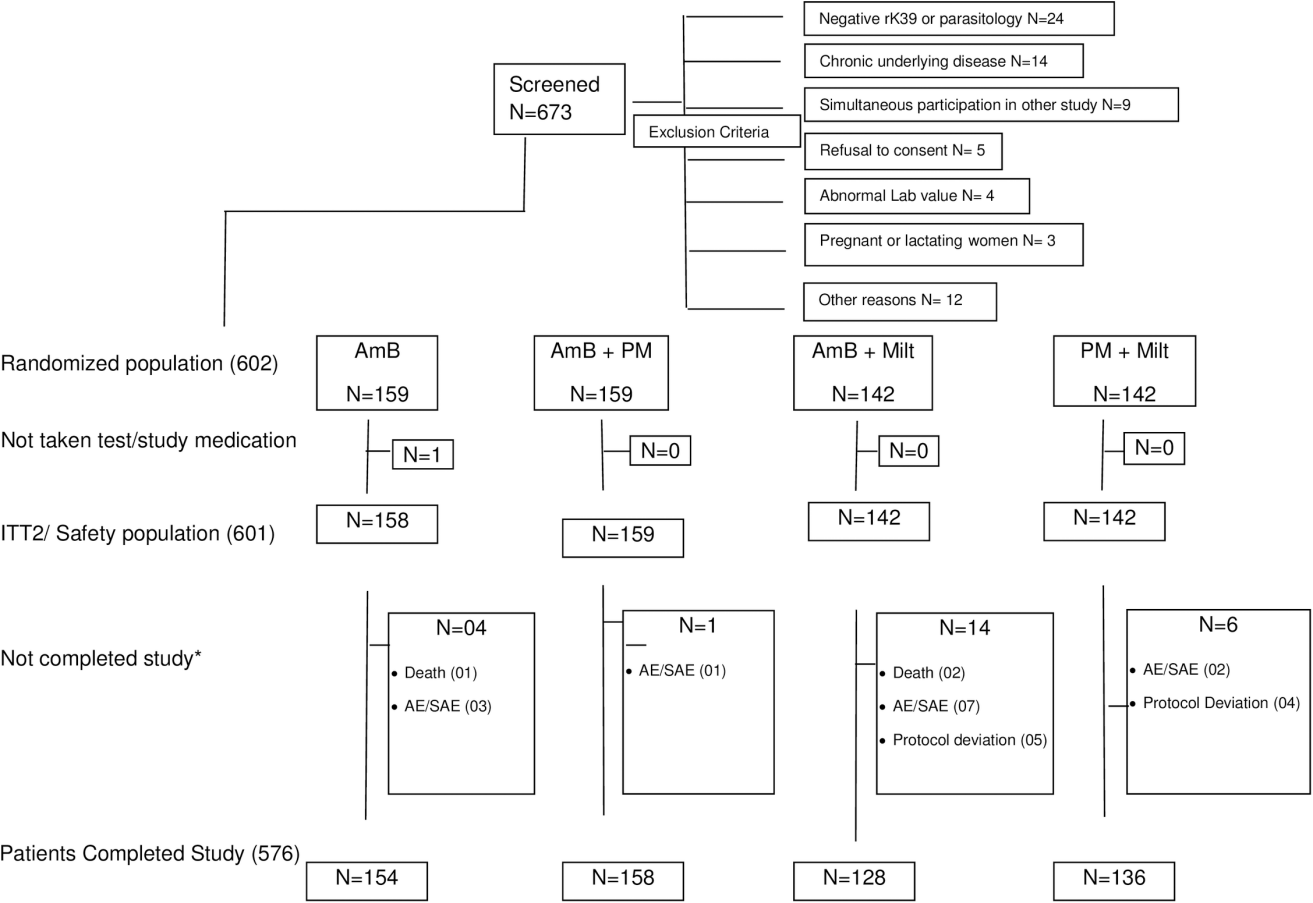
Patient disposition by treatment.

The patient flow through the study is shown in Fig 1. Patients were recruited from July 6, 2010 to September 2013 and asked to return to the clinic at day 45 and at 6 months after treatment onset, or sooner if any symptoms of VL reoccurred. Follow-up of all patients was completed by the end of March 2014. Randomisation produced groups with no significant differences in the main baseline characteristics across treatment groups (e.g. age, sex, weight, nutritional status, spleen size, haemoglobin) (Tables 2–4).

**Table 2:** Demographic characteristics by treatment arms (ITT population, N = 601).

Characteristics	Statistics	AmB 159	AmB + PM 159	AmB + Milt 142	PM + Milt 142	p value
**Age (years)**	N	158	159	142	142	0.1371^$^
	Mean (SD)	22.0 (14.50)	21.3 (14.33)	23.5 (14.59)	19.6 (13.49)	
	Min, Max	5, 55	5, 60	5, 60	5, 59	
**Height (cm)**	N	158	159	142	142	0.2593^$^
	Mean (SD)	145.0 (19.54)	143.3 (20.46)	146.3 (20.49)	141.8 (20.66)	
**Sex**						
**Male**	n (%)	93 (58.9)	88 (55.3)	98 (69.0)	95 (66.9)	0.0446^^^
**Female**	n (%)	65 (41.1)	71 (44.7)	44 (31.0)	47 (33.1)	
**Weight (kg)**	N	158	159	142	142	0.2827^$^
	Mean (SD)	35.39 (12.55)	34.53 (12.83)	36.86 (13.30)	34.04 (13.76)	

Denominator of the percentage is the number of patients in each treatment group.

^ p value is computed using chi-square test.

$ p value is computed using ANOVA.

One subject in the AmB arm who did not take at least one dose of study medication has been excluded from ITT Population.

**Table 3:** Baseline temperature, splenic characteristics and haemoglobin by treatment arms (ITT Population, N = 601).

Parameter (units)	Statistics	AmB	AmB + PM	AmB + Milt	PM + Milt
Axillary temperature (°F)	N	158	159	142	142
	Mean (SD)	99.95 (1.76)	99.74 (1.67)	99.66 (1.60)	99.57 (1.64)
Size of spleen* (cm)	N	158	159	142	142
	Mean (SD)	6.27 (4.41)	5.47 (3.60)	6.06 (3.91)	6.20 (3.65)
Haemoglobin (gm/dl)	N	158	158	142	142
	Mean (SD)	8.44 (1.432)	8.52 (1.545)	8.61 (1.406)	8.44 (1.358)

*Spleen size measured in the axial direction.

**Table 4:** Baseline clinical signs and symptoms by treatment arms (ITT population, N = 601).

**Clinical Signs**	**AmB N = 158 n (%)**	**AmB + PM N = 159 n (%)**	**AmB + Milt N = 142 n (%)**	**PM + Milt N = 142 n (%)**
Pallor	157 (99.4)	153 (96.2)	138 (97.2)	140 (98.6)
Icterus	14 (8.9)	7 (4.4)	5 (3.5)	9 (6.3)
Peripheral Lymphadenopathy	0	2 (1.3)	1 (0.7)	0
**Clinical Symptoms**	**AmB N = 158 n (%)**	**AmB + PM N = 158 n (%)**	**AmB + Milt N = 142 n (%)**	**PM + Milt N = 142 n (%)**
Feeling of weakness	156 (98.7)	156 (98.1)	141 (99.3)	141 (99.3)
Loss of weight	154 (97.5)	158 (99.4)	141 (99.3)	142 (100.0)
Anorexia	146 (92.4)	151 (95.0)	123 (86.6)	128 (90.1)
Nausea	103 (65.2)	112 (70.4)	92 (64.8)	94 (66.2)
Vomiting	32 (20.3)	32 (20.1)	28 (19.7)	19 (13.4)
Diarrhoea	5 (3.2)	5 (3.1)	8 (5.6)	5 (3.5)
Cough	72 (45.6)	66 (41.5)	68 (47.9)	55 (38.7)
Abdominal pain	102 (64.6)	97 (61.0)	89 (62.7)	83 (58.5)
Epistaxis	7 (4.4)	5 (3.1)	9 (6.3)	3 (2.1)

Denominator of the percentage is the number of patients in each treatment arm.

673 patients screened in the study, 602 randomised in the study, 71 patients did not meet inclusion criteria. 587 patients in the PP population, with: 3 deaths; 4 SAEs and 09 AEs that lead to treatment discontinuation, n = 13; 10 protocol deviations; 2 miltefosine redosing when vomiting occurred > 1h after administration; 3 patients missed a miltefosine dose; 2 received wrong Miltefosine capsule strength; 1 received additional miltefosine medication after finishing treatment; 1 mistake in randomization: randomized to PM+Milt administered AmBisome; 1 patient did not receive single dose of study medication after randomisation to AmBisome arm.

In the safety population (ITT) of 601 patients, 374 (62%) were males and 227 (38%) were females. 85% of patients had haemoglobin < 10 mg/dl at the time of screening. There were 594 (99%) patients who complained of weight-loss and feelings of weakness. There were 588 (98%) patients who had pallor at baseline.

The primary analysis was intention-to-treat; data were available for 158 patients randomized to AmBisome (1 withdrew consent before treatment started), 159 to AmBisome + paromomycin, 142 patients to AmBisome + miltefosine and 142 patients to paromomycin + miltefosine, with a total of 601 patients included in the ITT analysis.

Four patients were withdrawn from the study due to serious adverse events (SAEs), and another nine patients due to adverse events (AEs) that required treatment discontinuation, making a total of 13 early withdrawals due to adverse events. Three deaths occurred in the study: one in the AmBisome arm and two in AmBisome + miltefosine arm.

There were 10 cases of protocol deviations. Protocol deviations were due to miltefosine re-dosing to patients presenting with vomiting more than 1 hour after drug administration (2 patients), missing of a miltefosine dose (3 patients) or inadvertently receiving the wrong miltefosine capsule strength (2 patients). One patient took additional miltefosine after finishing her study medication, and one patient randomized to paromomycin + miltefosine treatment was administered AmBisome. Finally, one patient did not receive even a single dose of study medication after randomization to the AmBisome arm.

There were no patients lost to follow-up.

### Response to treatment

Cure rates at 45 days (initial cure) and 6 months (final cure) are shown in Tables 5 and 6.

**Table 5:** Definitive cure according to treatment arm and age groups.

Parameters	AmB	AmB + PM	AmB + Milt	PM + Milt
**Population Overall (ITT)**				
ITT	N = 158 n (%)	N = 159 n (%)	N = 142 n (%)	N = 142 n (%)
Definitive Cure at month 6	155 (98.1)	158 (99.4)	134 (94.4)	139 (97.9)
PP	N = 156 n (%)	N = 158 n (%)	N = 134 n (%)	N = 139 n (%)
Definitive Cure at month 6	154 (98.7)	158 (100.0)	128 (95.5)	136 (97.8)
**Population = Pediatrics (<12 yrs)**				
	N = 46 n (%)	N = 51 n (%)	N = 40 n (%)	N = 61 n (%)
Definitive cure at Month 6	46 (100.0)	51 (100.0)	40 (100.0)	59 (96.7)
**Population = Adolescents (≥12 to < 18 yrs)**				
	N = 33 n (%)	N = 32 n (%)	N = 20 n (%)	N = 14n (%)
Definitive cure at Month 6	32 (97.0)	32 (100.0)	19 (95.0)	14 (100.0)
**Population = Adults (≥18 yrs)**				
	N = 79 n (%)	N = 76 n (%)	N = 82 n (%)	N = 67 n (%)
Definitive cure at Month 6	77 (97.5)	75 (98.7)	75 (91.5)	66 (98.5)

Denominator of the percentage is the number of patients in each treatment group.

Definitive cure: Subjects with no treatment failure at Month 6.

**Table 6:** Initial cure according to treatment arms and age groups.

Population	AmB	AmB + PM	AmB + Milt	PM + Milt
ITT	N = 158 n (%)	N = 159 n (%)	N = 142 n (%)	N = 142 n (%)
Initial Cure at day 45	155 (98.1)	158 (99.4)	134 (94.4)	139 (97.9)
PP	N = 156 n (%)	N = 158 n (%)	N = 134 n (%)	N = 139 n (%)
Initial Cure at day 45	154 (98.7)	158 (100.0)	128 (95.5)	136 (97.8)
**Population = Pediatrics (<12 yrs)**				
	N = 46 n (%)	N = 51 n (%)	N = 40 n (%)	N = 61 n (%)
Initial cure at Day 45	46 (100.0)	51 (100.0)	40 (100.0)	59 (96.7)
**Population = Adolescents (≥12 to < 18 yrs)**				
	N = 33 n (%)	N = 32 n (%)	N = 20 n (%)	N = 14 n (%)
Initial cure at Day 45	32 (97.0)	32 (100.0)	19 (95.0)	14 (100.0)
**Population = Adults (≥18 yrs)**				
	N = 79 n (%)	N = 76 n (%)	N = 82 n (%)	N = 67 n (%)
Initial cure at Day 45	77 (97.5)	75 (98.7)	75 (91.5)	66 (98.5)

Denominator of the percentage is the number of patients in each treatment group.

Initial cure: subjects with no treatment failure at Day 45.

In the ITT population, the initial and final cure rates were >95% in all arms, except in the AmBisome + miltefosine arm (94.4% for both initial and final cure rate). All arms showed a final cure rate of >95% in the PP population.

In children, the initial cure rate is 100% in all groups except the paromomycin + miltefosine group with a cure rate of 96.7%. However, adolescents achieved 100% cure initial cure rate in the paromomycin + miltefosine group (Table 6).

In the ITT population, final cure rates were > 95% in children and adolescents in all groups. The lowest final cure rate was observed in the ITT population in adults treated with AmBisome + miltefosine (91.5%). Treatment failures in this arm were due to two cases of death not related to treatment and AEs or SAEs that lead to treatment discontinuation where patients required rescue treatment. Although not statistically significant, AmBisome + paromomycin was the most effective treatment with initial and final cure rates of 99.4% in the ITT population. Out of the first 120 patients that were included and followed up in the hospital setting (CBMC), parasitological cure measured on day 15 was achieved in > 90% of patients in all groups (patients who did not have cure confirmed were due to ‘no tissue to perform the test’ or ‘test not done’), and in 100% in the AmBisome + paromomycin group (Table 7). There were no relapses and no cases of PKDL up to 6 months follow-up. None of the combination treatments were inferior to AmBisome monotherapy when compared in pairs for both the intention-to-treat and the per-protocol populations (Table 1).

**Table 7:** Parasitological cure by treatment group.

**Study Visit**	**Parameters**	**AmB N = 32 n (%)**	**AmB + PM N = 32 n (%)**	AmB + Milt* N = 28 n (%)	**PM+ Milt N = 28 n (%)**
**ITT population**					
Screening	1+	9 (28.1)	12 (37.5)	8 (28.6)	12 (42.9)
	2+	8 (25.0)	10 (31.3)	8 (28.6)	3 (10.7)
	3+	6 (18.8)	6 (18.8)	2 (7.1)	6 (21.4)
	4+	8 (25.0)	4 (12.5)	10 (35.7)	6 (21.4)
	5+	1 (3.1)	0	0	1 (3.6)
Day 15	0	30 (93.8)	32 (100.0)	26 (92.9)	26 (92.9)
	No Tissue	1 (3.1)	0	1 (3.6)	1 (3.6)
	Not Done	1 (3.1)	0	0	1 (3.6)
**Study Visit**	**Parameters**	**AmB N = 32n (%)**	**AmB + PM N = 32 n (%)**	**AmB + Milt N = 27 n (%)**	**PM + Milt N = 28 n (%)**
**PP population**					
Screening	1+	9 (28.1)	12 (37.5)	8 (29.6)	12 (42.9)
	2+	8 (25.0)	10 (31.3)	8 (29.6)	3 (10.7)
	3+	6 (18.8)	6 (18.8)	2 (7.4)	6 (21.4)
	4+	8 (25.0)	4 (12.5)	9 (33.3)	6 (21.4)
	5+	1 (3.1)	0	0	1 (3.6)
Day 15	0	30 (93.8)	32 (100.0)	26 (96.3)	26 (92.9)
	No Tissue	1 (3.1)	0	1 (3.7)	1 (3.6)

Denominator of the percentage is the number of patients in each treatment group.

*One patient (AmB + Milt) died before Day 15.

### Safety and tolerability of treatments

There were 11 patients who experienced a total of 12 SAEs, which included three non-drug related deaths (severe pneumonia, sudden cardiac death and hepatic encephalopathy). In the miltefosine + paromomycin group, three drug-related SAEs occurred; two of which occurred in the same patient. This patient was a 50 year old male who developed drug induced nephropathy and ototoxicity two weeks after treatment, both probably related to paromomycin; mild hearing loss was still present at 6 months after treatment. In a 40 year old male, acute hepatitis developed and worsened during treatment but resolved spontaneously after treatment was finished. In the AmBisome + miltefosine group, a 35 year old female patient presented with high grade fever, rash and swelling of arms and legs after 2 days of Miltefosine, which was possibly drug related. Treatment was interrupted and she was later diagnosed with Rickettsial fever with concomitant nutritional oedema. Rescue treatment was given with AmBisome and she made a full recovery. None of the other non-fatal reported SAEs (encephalitis, internal bleeding due to a peptic ulcer, acute respiratory tract infection, epilepsy and viral encephalitis) were related to treatment.

368 out of 602 patients experienced at least one AE in the study. Approximately 34% of these AEs were related to treatment; these included vomiting in one fifth of patients in miltefosine containing treatment regimens and pyrexia in AmBisome containing treatment regimens. Vomiting directly after administration of miltefosine was common; there were 28 (20%) patients in the paromomycin + miltefosine arm and 16 (11%) in the AmBisome + miltefosine arm that needed re-dosing within the hour. The proportion of patients that experienced any treatment-related side effects was highest in the AmBisome + miltefosine arm (42%), and lowest (27%) in the AmBisome arm (Table 8).

**Table 8:** Most common adverse events (> 1%) related, probably or possibly related to treatment (ITT population).

	AmBisome N = 158	AmB + PM N = 159	AmB + Milt N = 142	PM + Milt N = 142
**Patients with at least one adverse event**	**43 (27%)**	**47 (30%)**	**60 (42%)**	**51 (36%)**
**Serious adverse events recorded**	**0**	**0**	**1**	**3**
**Discontinuation because of adverse events (related and unrelated)**	**3**	**1**	**7**	**2**
**Abdominal pain**	**0**	**0**	**3 (2%)**	**2 (1%)**
**Upper abdominal pain**	**0**	**2 (1%)**	**2 (1%)**	**0**
**Constipation**	**0**	**0**	**3 (2%)**	**4 (3%)**
**Diarrhoea**	**0**	**0**	**3 (2%)**	**4 (3%)**
**Nausea**	**0**	**0**	**4 (3%)**	**9 (6%)**
**Vomiting**	**0**	**2 (1%)**	**25 (18%)**	**27 (19%)**
**Injection site pain**	**0**	**5 (3%)**	**0**	**7 (5%)**
**Pain**	**0**	**0**	**2 (1%)**	**3 (2%)**
**Pyrexia**	**37 (23%)**	**35 (22%)**	**28 (18%)**	**9 (6%)**
**Jaundice**	**0**	**2 (1%)**	**0**	**0**
**Blood pressure increased**	**0**	**0**	**2 (1%)**	**0**
**Weight loss**	**0**	**0**	**0**	**2 (1%)**
**Anorexia**	**2 (1%)**	**0**	**3 (2%)**	**2 (1%)**
**Peripheral oedema**	**0**	**0**	**2 (1%)**	**0**
**Epistaxis**	**0**	**0**	**0**	**2 (1%)**
**Respiratory Distress**	**2 (1%)**	**0**	**2 (1%)**	**0**

Extensive biochemical testing was only done for patients treated in the hospital setting (120 patients). The clinically significant laboratory adverse events considered related to the study drug are described in Table 9. All of them were classified as mild.

**Table 9:** Laboratory adverse events related, probably or possibly related to treatment (ITT population) in CBMC (N = 120).

	AmBisome N = 32	AmB + PM N = 32	AmB + Milt N = 28	PM + Milt N = 28
**Increased bilirubin**	**0**	**1 (3.1%)**	**0**	**2 (7.1%)**
**Increased alanine aminotransferase**	**0**	**1 (3.1%)**	**1 (3.6%)**	**4 (14.3%)**
**Increased aspartate aminotransferase**	**0**	**1 (3.1%)**	**1 (3.6%)**	**4 (14.3%)**
**Prothrombin time prolonged**	**0**	**1 (3.1%)**	**0**	**0**

There were no statistically significant changes over the treatment period in any of the biochemical parameters. Haematological parameters (haemoglobin, red and white blood cell counts and platelets) were improved at day 45 as compared to baseline, without a significant difference between the individual treatment arms (S1–S4 Tables, supporting information).

## Discussion

All combinations proved non-inferior to the standard treatment with AmBisome, with definitive cure rate differences in relation to AmBisome compared to: AmBisome + paromomycin ITT 1.3% (95%CI -1.73, 4.27); AmBisome + miltefosine ITT1–3.7% (95%CI -9.20,1.85) and paromomycin + miltefosine ITT1–0.1% (95%CI -4.15, 4.03) (Table 1). Treatments were well tolerated and no new safety signal was identified. Overall adverse events were of mild intensity. The internationally accepted parameters for efficacy of VL treatment (≥95%) [8] were also met for all combinations in both the per-protocol (PP) and the intention-to-treat (ITT) populations, except for AmBisome + miltefosine, which showed an efficacy of 94.4% in the ITT population. These cases of failure were not due to lack of response or relapse, but related to two deaths and a higher number of treatment discontinuations in relation to adverse events, requiring rescue treatment. The trial was not powered to detect differences between the treatment arms; however, a small but significant difference was found between the efficacy of AmBisome + miltefosine and AmBisome + paromomycin in both the PP and ITT population. Post-hoc analysis of the data stratified per age group showed that this difference only remained significant in adult patients (Table 5). There was no loss to follow up at 6 months as patients were actively tracked by committed field workers, and there were no relapses or PKDL. However, there is recent evidence that most relapses occur after 6 months [9, 10]. We therefore recommend, in line with other authors [9], to follow VL patients for at least 12 months before determining the final treatment outcome.

The combination regimens described in this paper have been studied earlier in a Phase III clinical trial conducted in India (2008–2010) [7]. The excellent safety and efficacy outcomes in the present study support those found in India. The main difference with the Indian study is that patients have been mostly treated in field conditions at Upazila level, with treatment provided by government doctors. This study provides evidence that it is feasible to scale up the implementation of combination regimens within national program settings and that these are acceptable to patients as well as doctors. However, the patient population was selected to have non-severe disease, and we recommend active pharmacovigilance at sentinel sites in Bangladesh documenting treatment outcome (side effects, early treatment failure, relapse and PKDL) on the full patient population after implementation of combination regimens.

Recent discussions among decision-makers around the Road Map for elimination of VL in South Asia have led to the inclusion of single dose AmBisome as a first-line treatment and miltefosine + paromomycin as an alternative recommended treatment for VL in India and Bangladesh. Earlier, combination regimens had already been recommended by the WHO Expert Committee [8] and the Regional Technical Advisory Group (RTAG) for adoption by policy makers after demonstration of the feasibility of their implementation in field conditions [11]. The evidence generated in the present study supports the use of combination treatments as valid alternatives to single dose AmBisome therapy. The most cost effective combination appears to be 10 days of miltefosine + paromomycin, since this can be given on an outpatient basis [12]. However, ultimately the choice of treatment will depend on the circumstances. Considering the requirement for a cold chain for AmBisome and the availability of trained staff to give intravenous infusions, miltefosine and paromomycin given on outpatient basis may be a suitable treatment in most settings. But as miltefosine cannot be given to women of child bearing age who refuse contraception, AmBisome and paromomycin is a viable alternative. Given the fact that paromomycin is not currently registered in Bangladesh, AmBisome + miltefosine may be considered as an interim solution.

In Bangladesh, evidence on the excellent efficacy and safety profile of a single dose (10 mg/kg) of AmBisome was generated in 2010 [13] and it was soon thereafter adopted as a highly promising tool for regional elimination. Rolled out in a rural public hospital in Bangladesh, single dose AmBisome showed a final cure rate of 97% and this provided sufficient evidence for scaling up the use of AmBisome in the region as a first-line treatment in hospital settings [14]. Single dose AmBisome was adopted as a first-line treatment in Bangladesh in 2013, when the current clinical trial was still ongoing. Data from this clinical trial support the use of combination regimens as 2^nd^ line treatments for VL in Bangladesh.

The validation and use of combination therapy to provide an alternative to AmBisome in the context of a VL elimination program, where AmBisome is used as a first-line treatment for uncomplicated VL, relapse VL, HIV/VL co-infected patients and PKDL, is crucial.

## Supporting information

S1 Table Haemoglobin results and change from baseline by treatment group (ITT population, N = 601). (DOC)

S2 Table RBC count results and change from baseline by treatment group (CBMC, N = 120). (DOC)

S3 Table WBC count results and change from baseline by treatment group (CBMC, N = 120). (DOC)

S4 Table Platelet count results and change from baseline by treatment group (CBMC, N = 119). (DOC)
