# Supplementary material for: Safety and efficacy of short course combination regimens with AmBisome, miltefosine and paromomycin for the treatment of visceral leishmaniasis (VL) in Bangladesh
Source: PLoS Negl Trop Dis. 2017 May 30;11(5):e0005635. doi: 10.1371/journal.pntd.0005635 (PMC5466346; doi:10.1371/journal.pntd.0005635)
Supplement: S1 Table — (DOC) [file pntd.0005635.s001.doc]

|  |  |  | **AmBisome** | **AmB + PM** | **AmB + Milt** | **PM + Milt** |
| --- | --- | --- | --- | --- | --- | --- |
| **Hb (gm/dl)** |  |  |  |  |  |  |
|  | Screening | N= | 158 | 159 | 142 | 142 |
|  |  | Mean (SD) | 8.44 (1.432) | 8.55 (1.579) | 8.61 (1.406) | 8.44 (1.358) |
|  |  |  |  |  |  |  |
|  | Day 7 | N= | 157 | 158 | 141 | 142 |
|  |  | Mean (SD) | 8.54 (1.568) | 8.92 (1.563) | 8.97 (1.664) | 8.59 (1.470) |
|  | Change from baseline | N= | 157 | 158 | 141 | 142 |
|  |  | Mean (SD) | 0.10 (1.025) | 0.41 (1.159) | 0.35 (1.066) | 0.15 (1.143) |
|  | Day 15 | N= | 156 | 158 | 140 | 142 |
|  |  | Mean (SD) | 8.99 (1.646) | 9.52 (1.612) | 9.54 (1.654) | 9.40 (1.586) |
|  | Change from baseline | N= | 156 | 158 | 140 | 142 |
|  |  | Mean (SD) | 0.56 (1.346) | 1.01 (1.277) | 0.93 (1.331) | 0.96 (1.318) |
|  | Day 45 | N= | 156 | 158 | 140 | 142 |
|  |  | Mean (SD) | 10.01 (1.523) | 10.20 (1.480) | 10.25 (1.506) | 10.25 (1.402) |
|  | Change from baseline | N= | 156 | 158 | 140 | 142 |
|  |  | Mean (SD) | 1.58 (1.379) | 1.68 (1.387) | 1.65 (1.321) | 1.81 (1.534) |
|  | 6 months | N= | 156 | 158 | 140 | 142 |
|  |  | Mean (SD) | 10.91 (1.428) | 11.01 (1.300) | 11.24 (1.319) | 11.11 (1.333) |
|  | Change from baseline | N= | 156 | 158 | 140 | 142 |
|  |  | Mean (SD) | 2.48 (1.519) | 2.49 (1.422) | 2.63 (1.479) | 2.67 (1.525) |

**S1 Table: Haemoglobin results and change from baseline by treatment group (ITT population, N=601)**
