# Supplementary material for: Safety and efficacy of short course combination regimens with AmBisome, miltefosine and paromomycin for the treatment of visceral leishmaniasis (VL) in Bangladesh
Source: PLoS Negl Trop Dis. 2017 May 30;11(5):e0005635. doi: 10.1371/journal.pntd.0005635 (PMC5466346; doi:10.1371/journal.pntd.0005635)
Supplement: S2 Table — (DOC) [file pntd.0005635.s002.doc]

**S2 Table: RBC count results and change from baseline by treatment group (CBMC, N=120)**

|  |  |  | **AmBisome** | **AmB + PM** | **AmB + Milt** | **PM + Milt** |
| --- | --- | --- | --- | --- | --- | --- |
| **RBC**  **Million/cumm** |  |  |  |  |  |  |
|  | Screening | N= | 32 | 32 | 28 | 28 |
|  |  | Mean (SD) | 3.811(0.5921) | 3.848(0.6713) | 3.941(0.7162) | 3.896(0.6120) |
|  |  |  |  |  |  |  |
|  | Day 7 | N= | 31 | 32 | 27 | 28 |
|  |  | Mean (SD) | 3.885(0.5621) | 4.046(0.6301) | 4.323(0.7332) | 3.906(0.77) |
|  | Change from baseline | N= | 31 | 32 | 27 | 28 |
|  |  | Mean (SD) | 0.064(0.3756) | 0.197(0.3957) | 0.34(0.422) | 0.01(0.3554) |
|  | Day 15 | N= | 31 | 32 | 27 | 28 |
|  |  | Mean (SD) | 4.196(0.6422) | 4.265(0.6064) | 4.580(0.7227) | 4.443(0.6597) |
|  | Change from baseline | N= | 31 | 32 | 27 | 28 |
|  |  | Mean (SD) | 0.375(0.5243) | 0.417(0.4359) | 0.597(0.5655) | 0.547(0.3307) |
|  | Day 45 | N= | 31 | 32 | 27 | 28 |
|  |  | Mean (SD) | 4.429(0.4511) | 4.435(0.4926) | 4.647(0.7034) | 4.486(0.5451) |
|  | Change from baseline | N= | 31 | 32 | 27 | 28 |
|  |  | Mean (SD) | 0.608(0.4278) | 0.587(0.5135) | 0.664(0.6078) | 0.590(0.5709) |
|  | 6 months | N= | 31 | 32 | 27 | 28 |
|  |  | Mean (SD) | 4.622(0.3653) | 4.540(0.4425) | 4.687(0.5304) | 4.633(0.4931) |
|  | Change from baseline | N= | 31 | 32 | 27 | 28 |
|  |  | Mean (SD) | 0.801(0.5173) | 0.691(0.4979) | 0.704(0.6153) | 0.737(0.6360) |
