# Supplementary material for: Safety and efficacy of short course combination regimens with AmBisome, miltefosine and paromomycin for the treatment of visceral leishmaniasis (VL) in Bangladesh
Source: PLoS Negl Trop Dis. 2017 May 30;11(5):e0005635. doi: 10.1371/journal.pntd.0005635 (PMC5466346; doi:10.1371/journal.pntd.0005635)
Supplement: S3 Table — (DOC) [file pntd.0005635.s003.doc]

**S3 Table: WBC count results and change from baseline by treatment group (CBMC, N=120)**

|  |  |  | **AmBisome** | **AmB + PM** | **AmB + Milt** | **PM + Milt** |
| --- | --- | --- | --- | --- | --- | --- |
| **WBC**  **/cumm** |  |  |  |  |  |  |
|  | Screening | N= | 32 | 32 | 28 | 28 |
|  |  | Mean (SD) | 4556.3(2163.02) | 5009.4(1895.22) | 4182.1(1338.62) | 4221.4(1792.03) |
|  |  |  |  |  |  |  |
|  | Day 7 | N= | 31 | 32 | 27 | 28 |
|  |  | Mean (SD) | 6103.2(2473.93) | 5946.9(2318.92) | 6051.9(2054.33) | 5103.6(1850.42) |
|  | Change from baseline | N= | 31 | 32 | 27 | 28 |
|  |  | Mean (SD) | 1590.3(1895.5) | 937.5(2219.67) | 1807.4(1737.13) | 882.1(1570.1) |
|  | Day 15 | N= | 31 | 32 | 27 | 28 |
|  |  | Mean (SD) | 6393.5(2313.29) | 7003.1(2248.08) | 7325.9(2365.88) | 7521.4(2640.76) |
|  | Change from baseline | N= | 31 | 32 | 27 | 28 |
|  |  | Mean (SD) | 1880.6(2587.33) | 1993.8(2213.88) | 3081.5(2355.36) | 3300(2225.94) |
|  | Day 45 | N= | 31 | 32 | 27 | 28 |
|  |  | Mean (SD) | 7577.4(1975.64) | 8015.6(1751.15) | 7796.3(1951.23) | 8357.1(2091.50) |
|  | Change from baseline | N= | 31 | 32 | 27 | 28 |
|  |  | Mean (SD) | 3064.5(1983.52) | 3006.3(2272.69) | 3551.9(2310.24) | 4135.7(1785.38) |
|  | 6 months | N= | 31 | 32 | 27 | 28 |
|  |  | Mean (SD) | 8522.6(2415.05) | 9278.1(2671.93) | 8622.2(1954.15) | 8642.9(1938.2) |
|  | Change from baseline | N= | 31 | 32 | 27 | 28 |
|  |  | Mean (SD) | 4009.7(2322.98) | 4268.8(2949.62) | 4377.8(2353.94) | 4421.4(1946.57) |
