# Supplementary material for: Safety and efficacy of short course combination regimens with AmBisome, miltefosine and paromomycin for the treatment of visceral leishmaniasis (VL) in Bangladesh
Source: PLoS Negl Trop Dis. 2017 May 30;11(5):e0005635. doi: 10.1371/journal.pntd.0005635 (PMC5466346; doi:10.1371/journal.pntd.0005635)
Supplement: S4 Table — (DOC) [file pntd.0005635.s004.doc]

**S4 Table: Platelet count results and change from baseline by treatment group (CBMC, N=119)**

|  |  |  | **AmBisome** | **AmB + PM** | **AmB + Milt** | **PM + Milt** |
| --- | --- | --- | --- | --- | --- | --- |
| **Platelets**  **/cumm** |  |  |  |  |  |  |
|  | Screening | N= | 32 | 32 | 28 | 27* |
|  |  | Mean (SD) | 145,687.5 (58,725.41) | 156,687.5 (581,36.35) | 147,178.6 (624,52.98) | 144,296.3  (48,835.53) |
|  |  |  |  |  |  |  |
|  | Day 7 | N= | 31 | 32 | 27 | 27 |
|  |  | Mean (SD) | 220,322.6 (92,888.24) | 251,031.3 (95,776.11) | 217,111.1 (91,839.88) | 166,259.26  (78,099.34) |
|  | Change from baseline | N= | 31 | 32 | 27 | 27 |
|  |  | Mean (SD) | 75,225.8  (72,136.77) | 94,343.8 (81,415.15) | 66,518.5 (82,103.30) | 21,962.96  (73,911.84) |
|  | Day 15 | N= | 31 | 32 | 27 | 27 |
|  |  | Mean (SD) | 256,903.2 (90,802.48) | 278,125.0 (88,233.10) | 269,814.8 (90,090.57) | 323,518.52  (93,847.41) |
|  | Change from baseline | N= | 31 | 32 | 27 | 27 |
|  |  | Mean (SD) | 111,806.5 (90,219.52) | 121,437.5 (80,094.68) | 119,222.2 (76,532.21) | 179,222.22  (84,847.07) |
|  | Day 45 | N= | 31 | 32 | 27 | 27 |
|  |  | Mean (SD) | 238,064.5 (68,089.13) | 268,750.0 (75,498.34) | 252,222.2 (74,541.33) | 265,074.07  (84,851.42) |
|  | Change from baseline | N= | 31 | 32 | 27 | 27 |
|  |  | Mean (SD) | 92,967.7  (63,889.22) | 112,062.5 (78,090.08) | 101,629.6 (90,824.49) | 120,777.78  (60,255.44) |
|  | 6 months | N= | 31 | 32 | 27 | 27 |
|  |  | Mean (SD) | 228,709.7 (63,389.90) | 260,312.5 (69,118.15) | 250,185.2 (71,232.04) | 225,703.70  (62,131.26) |
|  | Change from baseline | N= | 31 | 32 | 27 | 27 |
|  |  | Mean (SD) | 83,612.9  (70,423.33) | 103,625.0 (87,190.10) | 99,592.6 (82,347.42) | 81,407.41  (60,395.66) |

* One subject was removed from the calculation of platelets parameter, due to not plausible value at baseline
